# Surface Decontamination on the Reconstructive Therapy of Peri‐Implantitis: A Multicenter Randomized Clinical Trial

**DOI:** 10.1111/cid.70075

**Published:** 2025-07-22

**Authors:** Alberto Monje, Sofía Navarro‐Mesa, Costanza Soldini, Giorgio Zappalá, Pedro Peña, Jose Manuel Navarro, Ramón Pons

**Affiliations:** ^1^ Department of Periodontology and Oral Medicine University of Michigan Ann Arbor Michigan USA; ^2^ Department of Periodontology Universitat Internacional de Catalunya Barcelona Spain; ^3^ Department of Periodontology University of Bern Bern Switzerland; ^4^ Private practice Branemark Clinic Las Palmas Spain; ^5^ Private practice Madrid Spain

**Keywords:** dental implant, endosseous implant complication, mucositis, peri‐implant disease, peri‐implantitis

## Abstract

**Objective:**

To compare the clinical/radiographic outcomes and the rate of disease resolution of the adjunctive use of electrolysis (GS) or hydrogen peroxide (HP) for mechanical decontamination in the reconstructive treatment of peri‐implantitis‐related intrabony defects.

**Material and Methods:**

A multicenter randomized clinical trial was designed to compare the effectiveness and safety of two strategies for the surface decontamination of crater‐like and circumferential intrabony defects subjected to reconstructive therapy. Clinical evaluation was made at baseline (T_0_), 6 months (T_1_) and 12 months (T_2_), while radiographic assessment was carried out at T_0_ and T_2_. Disease resolution was the primary outcome. Supportive therapy was administered following surgical treatment. Simple and multiple generalized estimating equations (GEE) models were applied to compare the outcomes achieved and to explore potential confounders. Post hoc power calculation was performed to validate the statistical power of the findings.

**Results:**

Overall, 58 patients completed the study. All the clinical parameters/indices, namely probing pocket depth, modified sulcular bleeding index, suppuration grading index, and width of keratinized mucosa, showed a significant reduction (*p* < 0.001) from T_0_ to T_2_ in both tested groups. Mucosal recession increased (*p* < 0.001) from T_0_ to T_2_. Marginal bone level and radiographic defect angle increased (*p* < 0.001) from T_0_ to T_2_. The disease resolution rate was 87.5% for the GS group and 64.5% for the HP group at T_2_ (*p* = 0.08). No major postoperative complications were reported.

**Conclusion:**

Both tested surface decontamination methods are effective in resolving peri‐implantitis, in gaining radiographic marginal bone levels, and in enhancing clinical peri‐implant conditions in the surgical reconstructive therapy (NCT05615051).

## 1 Introduction

The surgical treatment of peri‐implantitis is indicated when nonsurgical measures fail to resolve the inflammation, with the presence of residual pockets [1]. Reconstruction consists of using bone grafts and/or biologics with or without barrier membranes to gain marginal bone levels and reduce the pockets while enhancing the attachment level. Deep (≥ 3 mm) intrabony defects have been proposed as indicators for reconstructive therapy, due to the favorable prognosis associated with these scenarios [1]. It has been further suggested that defects exhibiting a contained morphology [2] with a narrow (≤ 40°) angular configuration [3] tend to result in consistent outcomes when reconstructive therapy is applied. Another highlighted key aspect for the success of this surgical strategy is surface decontamination. It is understood that the presence of residual biofilm and calculus, endotoxins, and bacterial colonies may compromise the outcome, leading to disease recurrence [4]. Consequently, the use of mechanical strategies and agents has been suggested in order to enhance the likelihood of achieving a relatively aseptic environment to promote peri‐implant health and re‐osseointegration. In this sense, it is important to remark that other mechanisms of peri‐implant inflammation have been proposed, including osteoimmune regulation underlying oral implant osseointegration, as well as the potential impact of titanium particulates and implant corrosion. These hypotheses would certainly modify the path implants are decontaminated [5, 6].

It has been consistently reported in the literature that single methods for surface decontamination are often inefficient [7, 8]. This may be explained by the topographic characteristics of modern dental implants, such as undercuts, grooves, and porosities that pose a drawback in the application of these strategies [4]. It is generally agreed that mechanical methods, such as titanium brushes, curettes, and ultrasonic devices, are needed to detach the biofilm from the implant surface [1]. In fact, this strategy has been seen to outperform other in vitro [9] and in vivo [10] methods in terms of surface decontamination and clinical outcomes, respectively. Nonetheless, titanium release from these instruments was found to be noticeable, and this, in turn, is linked to deleterious outcomes over the short term [11]. Traditional chemical and pharmacological methods have been further advocated to eliminate endotoxins from areas with limited access by mechanical tools. The level of evidence in this regard is low, and conclusions cannot be drawn regarding the superiority of any single method. Therefore, decision making on whether to use these traditional methods must rely on the perspective of the clinician and the mechanism of action.

Recently, the electrolytic method (GS) has been proposed to enhance effectiveness in decontaminating the infected implant surface [12, 13]. The implant must be loaded negatively with a voltage and a maximum current of 600 mA. GalvoSurge (Institut Straumann AG, Basel, Switzerland) produces hydrogen cations which penetrate the biofilm, and hydrogen bubbles form on the implant surface and disrupt the biofilm on the implant surface [12]. In vitro data have demonstrated the plausibility and effectiveness of this device [12, 14]. Nevertheless, clinical trials testing this novel system are limited. Single‐center studies have demonstrated that this strategy, combined with mechanical decontamination and compared with hydrogen peroxide (HP) likewise used as an adjunct to mechanical decontamination, is safe and effective in the reconstructive therapy of well‐contained peri‐implantitis‐related bone defects. Nevertheless, given the limited sample sizes involved, the outcomes were not solid, and caution was recommended in interpreting the data. Accordingly, the objective of this multicenter study was to compare the outcomes of these two strategies in a powered sample size.

## 2 Material and Methods

A multicenter, prospective, randomized, controlled two‐arm comparative study was conducted in accordance with the Declaration of Helsinki on human studies, following approval from the Ethics Committee of the University of Extremadura (Badajoz, Spain), the Ethics Committee of the International University of Catalonia (Barcelona, Spain), and the Ethics Committee of the University of Fernando Pessoa Canarias (Las Palmas de Gran Canaria, Spain). Patients were recruited from three centers: the CICOM‐MONJE Institute (Badajoz, Spain), the Drs. Pi & Esteller Clinic (Barcelona, Spain), and the Brånemark Center Las Palmas (Las Palmas de Gran Canaria, Spain). All patients received and signed a written informed consent form. Patient data was anonymized. The study was registered and approved at www.clinicaltrials.gov (NCT05615051), and is reported following the CONSORT statement guidelines [15].

### 2.1 Study Sample

An a priori calculation was carried out to determine the sample size with a statistical power to achieve significance at *p* < 0.05. A sample size of 50 patients was deemed suitable. To compensate for potential drop‐outs, 60 patients in total were determined to be recruited from the three centers. Quarterly reports between the investigators and coordinators (Nobel Biocare, Zurich, Switzerland) were scheduled during the study period to update on the evolution of the study. The following inclusion criteria were applied: patients aged 18–80 years, nonsmokers, with no infectious diseases at the time of implant placement or during the maintenance program, no systemic diseases or medications known to affect bone metabolism, and partially or completely edentulous individuals without active periodontal disease. Additionally, only peri‐implantitis bone defects where reconstructive therapy was indicated were included, specifically those with a contained defect configuration in implants positioned within the bony housing. Exclusion criteria included uncontained or combined peri‐implantitis‐related bone defects where reconstructive therapy was not viable, sites with less than 2 mm of keratinized mucosa on the buccal aspect, or implants located outside the bony housing based on intraoperative assessment. Furthermore, cement‐retained prostheses were excluded in cases where patients declined to sign the informed consent acknowledging the risk of prosthesis fracture during its retrieval.

### 2.2 Randomization

Patients were randomly assigned to either the test or control group, based on the last digit of their record number. This assignment was determined a priori by a nonblinded research assistant: a dental hygienist with experience in clinical research, who also assisted in data collection and management. Specifically, patients with record numbers ending in 0–4 were assigned to the test group, while those with record numbers ending in 5–9 were assigned to the control group. Once the target sample size for either group was reached at each center, subsequent patients were allocated exclusively to the remaining group to ensure completion of the total sample size.

### 2.3 Clinical Assessment

Peri‐implantitis was defined based on the 2017 World Workshop on Periodontal and Peri‐Implant Diseases, characterized by probing pocket depths of ≥ 6 mm and bone levels ≥ 3 mm apical to the most coronal portion of the intraosseous part of the implant, as determined from periapical radiographs [16]. For clinical assessment, intra‐examiner reliability was established prior to the study, requiring a k‐value of ≥ 0.85 (85% agreement) in 20% of the sample. A single clinical examiner per center (AM, RP, and SN) recorded all clinical variables at baseline and throughout follow‐up. The following clinical parameters and indices were assessed at T_0_ (5–6 weeks after nonsurgical therapy), T_1_ (6 months) and T_2_ (12 months). Due to significant travel restrictions related to the SARS‐CoV‐2 pandemic, inter‐examiner calibration could not be performed. The following parameters were reported:

- Pocket probing depth (PPD) recorded in millimeters using a plastic/metal North Carolina probe, applying an approximate probing force of 0.2 N.
- Modified sulcular bleeding index (mSBI) scored as 0–3 according to the extensiveness and severity of bleeding on probing.
- Mucosal recession (MR) was defined as the distance in millimeters from the implant–abutment connection as a steady mark and the mucosal margin.
- Keratinized mucosa (KM) around the dental implants, measured from the free mucosal margin to the mucogingival junction at the mid‐buccal position, to the nearest millimeter, using a North Carolina probe.
- Suppuration grading index (SGI) scored as 0–3 according to the extensiveness and severity of suppuration on probing.
- Intraoperative intrabony component (IC) measured intraoperatively at the mesial, medial, and distal aspects of the defect from the adjacent bony peak to the base of the defect using a North Carolina probe.

### 2.4 Outcomes

The primary outcome was the evaluation of disease resolution, while ancillary outcomes were the assessment of clinical and radiographic parameters to determine the impact of surface decontamination and other local factors (e.g., defect angle and depth) on the results at 12 months, as described elsewhere [17].

### 2.5 Radiographic Assessment

Periapical radiographs were obtained using the long cone paralleling technique, assisted by the intra‐oral radiographic positioning system. The radiographic variables were recorded at baseline (T_0_) and at the final follow‐up examination (T_2_) by a blinded examiner (RP) who calibrated the X‐rays based on the known thread distance and achieved an intraoperative *k*‐value ≥ 0.85 on a representative sample (20% of the total sample size) prior to commencing the study. The assessed radiographic parameters included marginal bone level (MBL) and the intra‐bony defect angle (DA).

### 2.6 Definition of Disease Resolution

Treatment success was assessed at the final evaluation. Peri‐implantitis was classified as “resolved” (R) if all of the following criteria were met:

- Absence of bleeding (BOP) and/or suppuration (SUP) upon gentle probing (~0.2 N).
- Probing pocket depths (PPD) ≤ 5 mm.
- No progressive radiographic bone loss beyond the standard error (SE ≥ 1 mm).

### 2.7 Therapeutic Modalities

**FIGURE 1:**
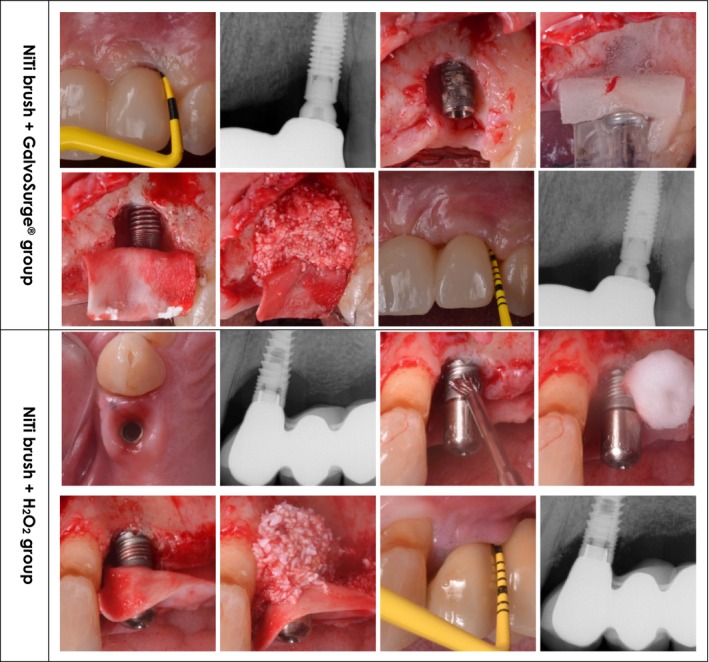
Clinical presentation and outcome for the groups included in the study.

Oral hygiene instructions were provided during the diagnostic phase. All eligible patients diagnosed with peri‐implantitis underwent nonsurgical therapy, performed by a single operator per center (AM, RP, and JMN), at least 5–6 weeks prior to the surgical reconstructive phase, as described elsewhere [17]. Concerning the surgical phase, a marginal internal bevel incision was performed to raise a full‐thickness flap, followed by debridement of granulation tissue using steel‐made curettes. Surface decontamination was then carried out using NiTi brushes (Hans Korea Co., Gyeonggi‐do, Korea) at 600 rpm for approximately 2–3 min. Subsequently, the following adjunctive decontamination strategies were applied:

- Test group (GS): GalvoSurge (Institut Straumann AG, Basel, Switzerland) for 2 min, followed by irrigation with saline solution (Figure 1).
- Control group (HP): Hydrogen peroxide (3%) for 2 min, followed by saline solution irrigation (Figure 1).

Reconstructive therapy involved collecting autogenous bone chips using a back‐action chisel, which were subsequently combined in a 1:1 ratio with anorganic bovine bone (creos xenogain, Nobel Biocare AB, Göteborg, Sweden). A collagen membrane (creos xenoprotect, Nobel Biocare AB, Göteborg, Sweden) was positioned over the defect, extending towards the buccal aspect in crater‐like defects. For implants in the esthetic zone, a subepithelial connective tissue graft was additionally placed on the buccal aspect, over the membrane, to prevent apical displacement of the mucosal margin (Schwarz et al. 2014). A similar distribution of implants in the anterior sites was allocated to the test and control groups. Closure was achieved using Nylon 5.0 sutures. All sites healed via a transmucosal (nonsubmerged) healing approach. Subsequently, patients were instructed to apply chlorhexidine and chitosan gel to the treated area three times daily for 2 weeks, in conjunction with a 7‐day course of systemic amoxicillin (750 mg, two tablets per day). Additionally, an antiinflammatory regimen of ibuprofen (600 mg, one tablet every 5–6 h for 5 days) was prescribed. Sutures were removed within 2–3 weeks, after which the patients were advised to resume their standard oral hygiene practices. All patients adhered to a structured 4‐month recall program for supportive peri‐implant maintenance therapy. Any complications arising during the early healing phase were systematically documented and reported.

### 2.8 Statistical Analysis

Descriptive statistical analyses were conducted for continuous (mean, standard deviation [SD], median, quartiles) and categorical variables (absolute/relative frequencies). Normality was assessed using the Kolmogorov‐Smirnov test and was confirmed for MBL, DA, and PPD, while other parameters required nonparametric methods. Binary logistic regression models estimated the probability of disease resolution from generalized estimated equations (GEE) subjected to the intra‐individual correlation. Unadjusted odds ratios (OR) and 95% confidence intervals (95% CI) were calculated via the Wald Chi^2^ test. A multiple binary regression model, including the variables that demonstrated significance, was conducted using the GEE. Model‐based estimators were calculated using exchangeable matrix correlations QIC goodness was calculated to fit statistics for the GEE model. Model validity was evaluated with diagnostic indicators and the ROC curve (AUC). For normally distributed parameters, the linear inter‐individual GEE model was estimated to assess and compare the outcomes along the follow‐up. Exchangeable correlation matrices were used, providing values in the range 0.33–0.64. To compare the treatment effects over time, the Bonferroni post hoc tests were applied. For nonnormal data, the Brunner–Langer model analyzed longitudinal changes using the ATS statistic and Wilcoxon (within‐group) and Mann–Whitney (between‐group) tests with Bonferroni correction. For MBL, mean values were calculated, and the GEE model using ANOVA linear model with mixed design was applied to assess the effect of MBL over time and Bonferroni test to compare the groups tested. Baseline group homogeneity was assessed with Chi^2^ and *t*‐test at the patient level and GEE models at the implant level. A post hoc analysis confirmed 80.6% power to detect a significant difference in resolution rates (60% vs. 90%) at a 95% confidence level (*α* = 0.05). The software used were R 4.3.1 (R Core Team, 2023), R: A language and environment for statistical computing, R Foundation for Statistical Computing, Vienna, Austria. URL http://www.R‐project.org/ and SPSS 15.0 (Chicago, IL, USA).

## 3 Results

Consecutive patients diagnosed with peri‐implantitis were recruited and evaluated from January 2022 to December 2023. Overall, 58 patients completed the study; two dropouts were reported due to incidents that precluded their attendance to supportive peri‐implant therapy (Table 1). Of these subjects, 37 were women (63.8%) and 21 men (36.2%), with an overall mean age of 62.4 ± 10.7 years (range 33–88 years). In total, 5 of the patients contributed two implants to the study and the remaining 53 only one implant. Hence, the distribution of the groups was as follows: HP group: *n*_patients_ = 29, *n*_implants_ = 31 and GS group: *n*_patients_ = 29, *n*_implants_ = 32. Of these, 2 were tissue‐level, while the remaining 61 were bone‐level implants. Regarding surface topographic features, 48 were TiUnite (Nobel Biocare AB, Göteborg, Sweden), 4 titanium‐plasma spray (Institut Straumann AG, Basel, Switzerland), 4 TiOblast (Astra Tech, Mölndal, Sweden), 3 resorbable blast media (TICARE, Valladolid, Spain), 2 sandblasted, large‐grit, acid‐etched (Institut Straumann AG, Basel, Switzerland), 1 Laser‐Lok (BioHorizons, Birmingham, USA), and 1 NanoTec (Alpha Bio Tec., Petah Tikva, Israel). Implant positions were 46% molars, 33.3% premolars, and 20.6% incisors. In turn, 55.6% of the implants were located in the maxilla, while 44.4% were located in the mandible. Mean intrabony defect depth was 3.98 ± 0.91 mm, with 46% being classified as Ib and 54% as Ic [18]. Homogeneity of both groups for the demographic, clinical setting, radiographic, and clinical data was according to the results of the Chi^2^ test and the two‐sample *t*‐test for patient‐level variables and GEE models for the analyses at implant‐level.

**TABLE 1:** Demographic, clinical, and radiographical characteristics of the patients and implants included in the study at baseline: *N* (%)/mean ± SD; *p*‐value from Chi^2^, Fisher's exact test, 2‐sample *t*‐test at patient level, and Wald Chi^2^ of GEE model at implant level.

	HP	GS	*p*
Total patients	29 (100)	29 (100)	
Gender			0.785 (Chi^2^)
Male	10 (34.5)	11 (37.9)	
Female	19 (65.5)	18 (62.1)	
Age	61.8 ± 9.8	63.1 ± 11.7	0.645 (t)
Implants (*n*)			1 (Fis)
1	27 (93.1)	26 (89.7)	
2	2 (6.9)	3 (10.3)	
Total implants	31 (100)	32 (100)	
Position			0.092 (Chi^2^)
Anterior	7 (22.6)	6 (18.8)	
Premolar	14 (45.2)	7 (21.9)	
Molar	10 (32.3)	19 (59.4)	
Arch			0.186 (Chi^2^)
Maxilla	20 (64.5)	15 (46.9)	
Mandible	11 (35.5)	17 (53.1)	
Defect depth	4.19 ± 1.01	3.78 ± 0.75	0.06 (Chi^2^)
Morphology			0.416 (Chi^2^)
Ib	16 (51.6)	13 (40.6)	
Ic	15 (48.4)	19 (59.4)	
Pocket probing depth (mm)	6.27 ± 1.26	5.76 ± 1.02	0.154 (Chi^2^)
Modified sulcular bleeding index	1.53 ± 0.74	1.24 ± 0.78	0.566 (Chi^2^)
Suppuration grading index	0.43 ± 0.57	0.52 ± 0.70	0.758 (Chi^2^)
Keratinized mucosa (mm)	3.73 ± 1.36	3.83 ± 1.24	0.758 (Chi^2^)
Mucosal recession (mm)	−1.00 ± 0.89	−0.97 ± 1.26	0.912 (Chi^2^)
Marginal bone loss (mm)	4.45 ± 0.83	4.16 ± 1.02	0.462 (Chi^2^)
Defect angle (°)	34.56 ± 8.69	33.02 ± 8.05	0.494 (Chi^2^)

### 3.1 Clinical Outcomes

**FIGURE 2:**
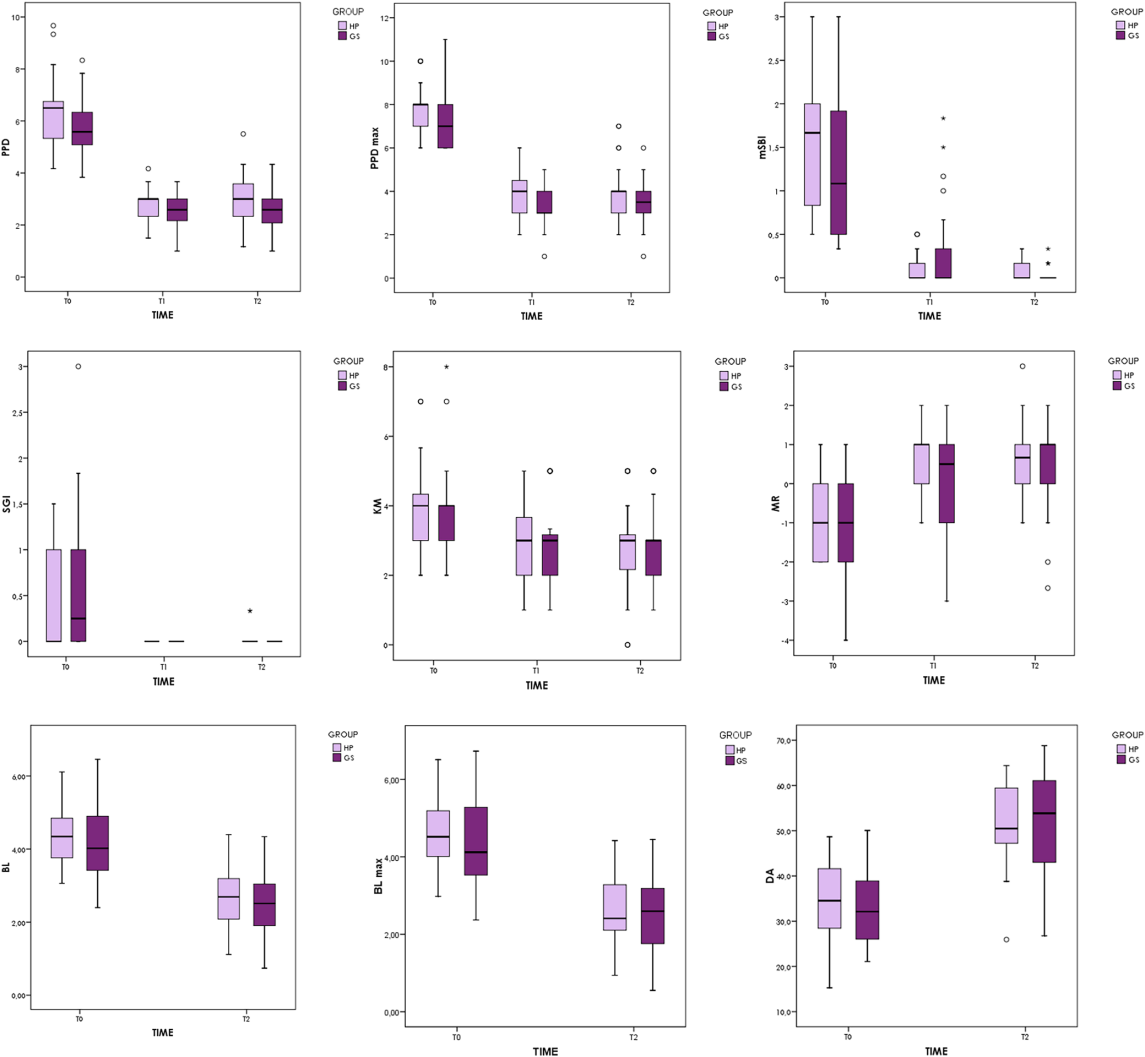
Clinical and radiographic parameters at the different follow‐up assessments.

Mean PPD reduction from T_0_ to T_2_ was 3.15 mm for GS and 3.31 mm for HP, reaching statistical significance (Wald Chi^2^ GEE = 341.9; df = 1; *p* < 0.001) for both groups. Nonetheless, from T_1_ to T_2_, no significant differences were observed. Moreover, no differences were noticed between the evaluated groups at T_2_ (Wald Chi^2^ GEE = 2.06; df = 1; *p* = 0.152). Mean reduction at deepest PPD from T_0_ to T_2_ was 4 mm, with no notable differences between the tested groups (Wald Chi^2^ GEE = 1.51; df = 1; *p* = 0.217). With regard to mSBI, the median reduction between T_0_ and T_2_ was −1.08 and −1.67 for the GS and HP groups, respectively. Statistical significance was reached from T_0_ to T_2_ (Wilcoxon's Z = 4.95; *p* < 0.001) and from T_1_ to T_2_ (Wilcoxon's *Z* = 2.77; *p* = 0.018). Mean mSBI at T_2_ was 0.07 for HP and 0.03 for GS. No statistically significant difference was noticed between the evaluated groups (Mann–Whitney's *Z* = 2.08; *p* = 0.112). Likewise, for SGI, statistical significance was reached from T_0_ to T_2_ (Wilcoxon's *Z* = 3.74; *p* < 0.001). No difference was recorded in this parameter between the evaluated groups (Mann–Whitney's *Z* = 1.45; *p* = 0.442). At T_2_, however, mean SGI was 0.02 for HP and 0 for GS. Interestingly, a median reduction of 1 mm of buccal KM was recorded for both groups from T_0_ to T_2_ (Wilcoxon's *Z* = 4.05; *p* < 0.001 for GS; Wilcoxon's *Z* = 3.62; *p* < 0.001 for HP). There were no differences between the evaluated groups. On the other hand, MR consistently increased by 1 mm in both groups from T_0_ to T_2_ (Wilcoxon's *Z* = 4.81; *p* < 0.001 for GS; Wilcoxon's *Z* = 4.14; *p* < 0.001 for HP), with no statistically significant difference between them (Figure 2).

### 3.2 Radiographic Outcomes

Marginal bone level increased 1.61 ± 0.75 mm for the GS group and 1.66 ± 0.58 mm for the HP group from T_0_ to T_2_. Mean gain for deepest MBL was 1.88 ± 0.82 and 1.87 ± 0.62 mm for the GS and HP groups, respectively. Variations were noticed during the study period (test GLM = 349.7; df = 1; *p* < 0.001), but there were no significant differences between groups (*F* test GLM = 0.08; df = 1; *p* = 0.785). Regarding DA, an increase was recorded in both groups (HP = 19.3°, GS = 17.6°). Variations were observed during the study period (test GLM = 311.5; df = 1; *p* < 0.001), but there were no significant differences between groups (*F* test GLM = 0.22; df = 1; *p* = 0.638).

### 3.3 Disease Resolution

**FIGURE 3:**
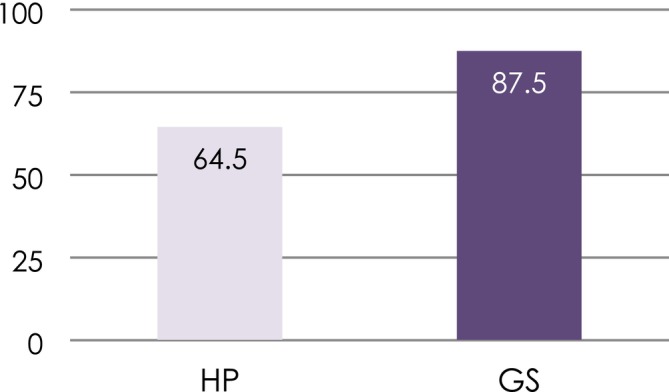
Disease resolution rate for the evaluated decontamination modalities.

**FIGURE 4:**
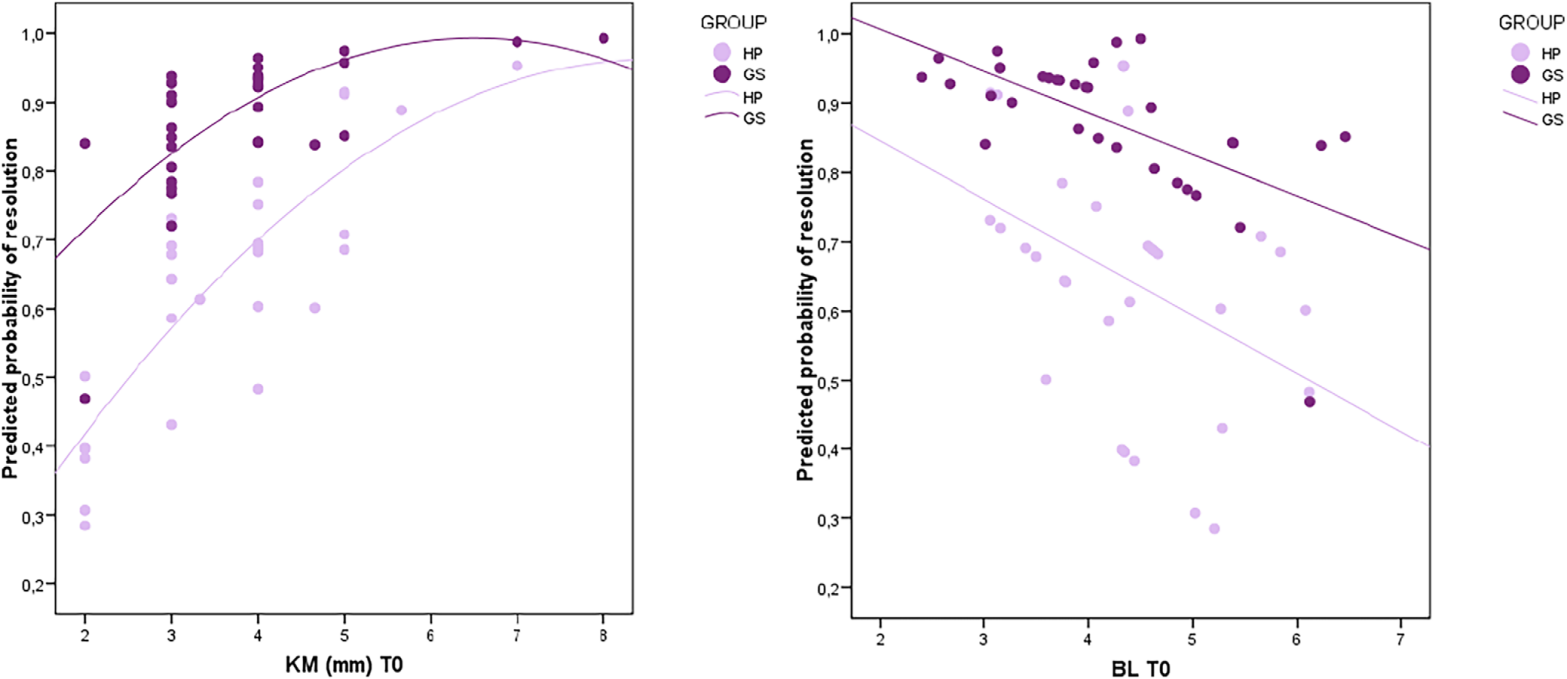
Probability for disease resolution according to (a) the band of keratinized mucosa and (b) marginal bone loss.

The disease resolution rate was 87.5% for the GS group and 64.5% for the HP group at T_2_ (Figure 3). The nonadjusted model supported the influence of the surface decontamination modality on disease resolution at T_2_ (Wald Chi^2^ GEE = 5.01; df = 1; OR = 5.09; *p* = 0.078) without achieving statistical significance. Significance, however, was observed between the width of KM at baseline and disease resolution (Wald Chi^2^ GEE = 4.23; df = 1; OR = 1.85; *p* = 0.040). Interestingly, baseline MBL also showed a tendency towards significance of resolution (Wald Chi^2^ GEE = 3.33; df = 1; OR = 0.64; *p* = 0.068). The adjusted model confirmed the significance between the width of KM (Wald Chi^2^ GEE = 6.89; df = 1; OR = 2.21; 95% CI: 1.19–3.34; *p* = 0.009) and marginal bone level at baseline (Wald Chi^2^ GEE = 4.18; df = 1; OR = 0.56; 95% CI: 0.33–0.97; *p* = 0.041) and disease resolution (Figure 4). The use of GS demonstrated a tendency towards the achievement of statistical significance with disease resolution (Wald Chi^2^ GEE = 2.91; df = 1; OR = 3.76; 95% CI: 0.75–18.8; *p* = 0.088). None of the other variables explored demonstrated significance. The QIC goodness indicator was 64.8%, representing an enhanced 10% when compared to the nonadjusted model. The logistic model equation derived from this finding is as follows:

$$\frac{p}{1-p}=2.01\;3.76^{\mathrm{GS}}\;2.21^{\mathrm{KM}}\;0.56^{\mathrm{BL}}$$

Considering these three parameters (MBL, surface decontamination modality and KM), 76.2% of the cases could be accurately classified as resolved or nonresolved. The sensitivity and specificity, therefore, were 91.7% and 26.7%, respectively. The area under the curve (AUC) was 77.9% (95% CI: 65.8%–90%; *p* = 0.001, Figure S4).

### 3.4 Postoperative Complications

Overall, 64.2% of the implant sites healed uneventfully, while 35.8% experienced some form of complication. The most common complication was mucosal dehiscence during the early healing phase (< 21 days), affecting 31.7% of the implants. Additionally, in three of these cases (4.7%), mucosal dehiscence was associated with exudate. The second most frequent complication was moderate to severe pain, reported by 11 patients (19%), including 6 from the control group and 5 from the GS group, during the first 5–7 days of healing. However, the pain had gradually subsided by the time of the suture removal visit. Furthermore, three sites (4.7%) experienced hemorrhage between days 2 and 4, which was effectively managed with tranexamic acid‐soaked gauze and cyanoacrylate. Additionally, two patients from the GS group (3.4%) and one from the control group (1.7%) developed severe hematomas in the maxilla or mandible, which resolved between days 10 and 14 post‐treatment. Lastly, in three patients (4.7%), the healing abutments loosened within the first 10 days after treatment and required re‐tightening. No permanent sensory disturbances or additional complications beyond those mentioned were reported.

## 4 Discussion

Surface decontamination in the treatment of peri‐implantitis remains a critical challenge for enhancing therapeutic success, particularly in patients subjected to reconstructive treatments. A wide range of methods and agents have been explored and documented in the literature [7, 19]. Interestingly, to date the use of chemicals or photodynamic therapy has not yielded superior outcomes compared to mechanical debridement [8]. On the other hand, it is acknowledged that owing to the surface topographic characteristics, the use of mechanical methods may result insufficient to destroy the organic components of the bacteria, eliminate their lipopolysaccharides, and reduce the microbial colonies. On the other hand, the use of such agents might be associated with structural and chemical modifications and changes in wettability and hydraulicity of the implant surface that may deleteriously affect subsequent homeostasis and ultimately peri‐implant health [20]. For years, the use of HP as an adjunct to mechanical tools was based on its antimicrobial activity through the production of reactive oxygen species (ROS). This combination was shown to be effective in treating peri‐implantitis [10, 21, 22, 23]. However, despite its superiority to other strategies as established by in vitro studies, the effectiveness of this approach in suppressing bacterial regrowth is suboptimal in terms of facilitating re‐osseointegration [24]. In this regard, emerging technologies were proposed to overcome the limitations associated with the traditional methods. Electrolysis (GS) was suggested as a minimally invasive approach to remove the microorganisms adhered to the implant surface [25]. Clinical and case studies demonstrated the safety and effectiveness of this strategy [14, 26, 27, 28]. Single‐center outcomes from the present multicenter randomized clinical trial have been presented elsewhere [17] and have validated the effectiveness of GS in treating peri‐implantitis‐related intrabony defects. However, due to the limited sample sizes involved, robust conclusions could not be drawn. Findings from the present multicenter study thus allow a more in‐depth assessment of the superiority of the method. Interestingly, the disease resolution rate was seen to be higher (~4×) for GS compared to HP, showing a tendency towards statistical significance. This finding is attributed to the composite definition of disease resolution that included no bleeding on probing and no suppuration. Given that mSBI and SGI were higher at the HP group, disease resolution proved higher for GS. These findings might be attributed to the higher level of hard and soft tissue homeostasis and adaptation following the upgraded decontamination process using GS in contrast to the suboptimal effectiveness of HP to decontaminate the surface. This would translate to a higher mass of residual biofilm attached to the implant surface that may compromise the long‐term outcomes. Hence, the evaluation of long‐term stability following reconstructive therapy with these two surface decontamination strategies is advisable in order to assess the sustainability of the outcomes.

No clear superiority has been proven for any of the surface decontamination strategies reported in the literature to date [4]. Considering that hard deposits must be detached from the implant surface, it seems reasonable to recommend mechanical strategies [4]. In the case of firmly adhered calcified deposits, titanium brushes may be a valid option [29]. In vitro studies [30, 31] have pointed out that this type of mechanical instrument outperforms other tools in intrabony components, since its flexibility offers better access to the thread valleys. In vivo research [10] has demonstrated an increased disease resolution rate and radiographic bone fill when compared to the use of plastic ultrasonic scalers. However, it is important to disclose the potential impact of titanium particulates released using mechanical instruments [11] that may alter key inflammatory cascades in the peri‐implant tissues including toll‐like receptor activation and inflammasome and complement signaling, which lead to nonresolving destructive inflammation (i.e., disease progression/resolution) [32]. In particular, the use of titanium brushes demonstrated a reduced corrosion resistance and increased titanium, while titanium particles exerted cytotoxic effects against fibroblasts and reduced osteoconductivity [33]. The use of chemical and pharmacological agents has been advocated to eliminate the lipopolysaccharides and reduce bacterial colonies in order to inhibit collagenase activity and minimize apoptotic activity while also eliciting antiinflammatory effects [34, 35]. HP, in particular, has shown effectiveness in reducing 92% of the bacteria in an oral biofilm model [36] by generating hydroxyl radicals [37]. Histological evidence of re‐osseointegration after decontamination with 10% HP has been obtained in two animal studies [38, 39]. Several clinical trials have demonstrated the effectiveness of HP in decontaminating the implant surface [10, 40, 41, 42, 43, 44, 45]. Findings from the present study showed that disease resolution was consistently achieved in 64.5% of the treated cases. Therefore, it seems that the use of this chemical as an adjunct to mechanical decontamination results in favorable outcomes. Nevertheless, GS outperformed HP in terms of disease resolution, without reaching statistical significance. This can be attributed to the mechanism of action of this technology, where a sodium formate solution acting as an electrolyte is pumped by a device through a platinized ring acting as an anode and sprayed on the exposed and infected implant surface [46]. GS produces hydrogen cations which penetrate the biofilm, and the hydrogen bubbles emerge on the implant surface to disrupt the biofilm on the implant surface. In vitro findings have suggested that this approach is effective in removing bacteria in biofilm models [35, 47]. Nevertheless, it seems that tissue cell morphology and integrity might be impaired with the use of this method, which may compromise cell cytocompatibility [35]. Future studies are warranted to shed light on the effect of GS on osteoblast behavior.

Different biomaterials have been used in the reconstructive treatment of peri‐implantitis [48]. No differences have been reported in terms of regenerative outcomes, except for the use of autogenous bone alone [49]. We opted to use a mixture of autogenous and anorganic bovine bone in a 1:1 ratio. It is known that the use of autogenous bone chips involves a release of growth factors such as TGF‐β1 and BMP‐2, both of which are involved in osteogenesis. On the other hand, anorganic bone particles provide scaffolding and osteoconductivity [50]. This mixture has demonstrated successful outcomes in alveolar bone regeneration [51]. The present study validated this mixture for the treatment of intrabony defects.

Interestingly, 64.2% of the implant sites healed uneventfully, while 35.8% experienced some form of complication. The most common problem was mucosal dehiscence during the early healing phase (< 21 days), affecting 31.7% of the implants. A recent study [52] has reported similar complication rates: soft tissue dehiscence occurred in 19% of the cases, exposure of the membrane in 9.5%, and exposure of the bone substitute in 4.8%. In fact, the use of the barrier membrane increases the incidence of postoperative complications [52], without adding any further benefit to the reconstructive outcomes of well‐contained defects [21]. Given the relatively high rate of mucosal dehiscence, it might be advisable to limit the use of barrier membranes to partially‐contained defects. On the other hand, the rationale for using barrier membranes is based on compartmentalization and the up‐regulation of genes for bone healing at the interface membrane/defect such as osteocalcin, bone‐morphogenetic protein‐2, cathepsin‐K and RANKL [53]. Moreover, it has been speculated that because of the mechanism of action of GS, neurosensory complications may be frequent. Severe pain was reported in 19% of the cases, being very similar with both approaches used for surface decontamination. In this sense, it might be advisable to apply block anesthesia when using GalvoSurge, in order to minimize temporary sensory disturbances. In this sense, in the future, patient‐reported outcomes are advisable being reported to better understand patients´ preference towards the different tested strategies.

The findings from this study must be interpreted with caution due to limitations associated with the design involved. First of all, case selection was limited to intrabony defects of implants inside the bony housing. Cases exhibiting compartments outside the bony housing might not respond favorably to this therapeutic modality, and other measures, such as implantoplasty, might be advisable to minimize bacterial recolonization of the exposed implant surface [54]. In addition, despite the use of a positioning system to maximize the reliability of the X‐rays, a personalized occlusal registration key was not used. Moreover, patient‐reported outcome measures would have been of benefit for understanding the patient preferences. On the other hand, the sample size and low dropout rate constitute major strengths of this study. It is important to underscore that these favorable findings for both evaluated groups also evidence the importance of supportive maintenance for peri‐implant health. Long‐term assessments will help to validate the effectiveness of these two methods for surface decontamination. Moreover, it might be convenient in the future to compare in vivo mechanical strategies combined with GS to mechanical strategies alone to shed light on the role of the GS in surface decontamination.

## 5 Conclusions

Both tested surface decontamination methods are effective in resolving peri‐implantitis, in gaining radiographic marginal bone levels, and in enhancing clinical peri‐implant conditions in the surgical reconstructive therapy. The use of GS as an adjunct to mechanical surface decontamination results ~4× more effective in achieving disease resolution in the reconstructive treatment of peri‐implantitis‐related intrabony bone defects when compared to HP.

## Author Contributions

A.M. conceived the study. All the authors participated equally in data acquisition.

## Conflicts of Interest

A.M. and P.P. disclose receiving fees for lecturing and other education‐related events from Nobel Biocare (Zurich, Switzerland) and Straumann (Basel, Switzerland). None of the authors have any direct conflicts of interest with any of the instruments/materials listed in this manuscript.

## Supporting information


Data S1.

Data S2.

Data S3.

Data S4.

## Data Availability

The data that support the findings of this study are available from the corresponding author upon reasonable request.

## References

[1] D. Herrera , T. Berglundh , F. Schwarz , et al., “Prevention and Treatment of Peri‐Implant Diseases‐The EFP S3 Level Clinical Practice Guideline,” Journal of Clinical Periodontology 50, no. S26 (2023): 4–76, 10.1111/jcpe.13823. 37271498

[2] F. Schwarz , M. Herten , M. Sager , K. Bieling , A. Sculean , and J. Becker , “Comparison of Naturally Occurring and Ligature‐Induced Peri‐Implantitis Bone Defects in Humans and Dogs,” Clinical Oral Implants Research 18, no. 2 (2007): 161–170, 10.1111/j.1600-0501.2006.01320.x. 17348880

[3] A. Monje , R. Pons , A. Sculean , J. Nart , and H. L. Wang , “Defect Angle as Prognostic Indicator in the Reconstructive Therapy of Peri‐Implantitis,” Clinical Implant Dentistry and Related Research 25, no. 6 (2023): 992–999, 10.1111/cid.13244. 37405662

[4] A. Monje , E. Amerio , J. K. Cha , et al., “Strategies for Implant Surface Decontamination in Peri‐Implantitis Therapy,” International Journal of Oral Implantology 15, no. 3 (2022): 213–248. 36082658

[5] T. Albrektsson , P. Tengvall , L. Amengual , P. Coli , G. A. Kotsakis , and D. Cochran , “Osteoimmune Regulation Underlies Oral Implant Osseointegration and Its Perturbation,” Frontiers in Immunology 13 (2022): 1056914, 10.3389/fimmu.2022.1056914. 36761175 PMC9902598

[6] G. A. Kotsakis and D. G. Olmedo , “Peri‐Implantitis Is Not Periodontitis: Scientific Discoveries Shed Light on Microbiome‐Biomaterial Interactions That May Determine Disease Phenotype,” Periodontology 2000 86, no. 1 (2021): 231–240, 10.1111/prd.12372. 33690947

[7] A. Wilensky , L. Shapira , A. Limones , and C. Martin , “The Efficacy of Implant Surface Decontamination Using Chemicals During Surgical Treatment of Peri‐Implantitis: A Systematic Review and Meta‐Analysis,” Journal of Clinical Periodontology 50, no. S26 (2023): 336–358, 10.1111/jcpe.13794. 36792071

[8] I. Hart , C. Wells , A. Tsigarida , and B. Bezerra , “Effectiveness of Mechanical and Chemical Decontamination Methods for the Treatment of Dental Implant Surfaces Affected by Peri‐Implantitis: A Systematic Review and Meta‐Analysis,” Clinical and Experimental Dental Research 10, no. 1 (2024): e839, 10.1002/cre2.839. 38345466 PMC10847712

[9] Y. Ichioka , L. Virto , P. Nuevo , et al., “Decontamination of Biofilm‐Contaminated Implant Surfaces: An In Vitro Evaluation,” Clinical Oral Implants Research 34, no. 10 (2023): 1058–1072, 10.1111/clr.14136. 37469250

[10] B. de Tapia , C. Valles , T. Ribeiro‐Amaral , et al., “The Adjunctive Effect of a Titanium Brush in Implant Surface Decontamination at Peri‐Implantitis Surgical Regenerative Interventions: A Randomized Controlled Clinical Trial,” Journal of Clinical Periodontology 46, no. 5 (2019): 586–596, 10.1111/jcpe.13095. 30825341

[11] D. Daubert , E. Lee , A. Botto , M. Eftekhar , A. Palaiologou , and G. A. Kotsakis , “Assessment of Titanium Release Following Non‐Surgical Peri‐Implantitis Treatment: A Randomized Clinical Trial,” Journal of Periodontology 94, no. 9 (2023): 1122–1132, 10.1002/JPER.22-0716. 37070363 PMC10524263

[12] C. Ratka , P. Weigl , D. Henrich , F. Koch , M. Schlee , and H. Zipprich , “The Effect of In Vitro Electrolytic Cleaning on Biofilm‐Contaminated Implant Surfaces,” Journal of Clinical Medicine 8, no. 9 (2019): 1397, 10.3390/jcm8091397. 31500093 PMC6780638

[13] M. Stiesch , J. Grischke , P. Schaefer , and L. J. A. Heitz‐Mayfield , “Supportive Care for the Prevention of Disease Recurrence/Progression Following Peri‐Implantitis Treatment: A Systematic Review,” Journal of Clinical Periodontology 50, no. S26 (2023): 113–134, 10.1111/jcpe.13822. 37339881

[14] H. Zipprich , P. Weigl , R. Di Gianfilippo , et al., “Comparison of Decontamination Efficacy of Two Electrolyte Cleaning Methods to Diode Laser, Plasma, and Air‐Abrasive Devices,” Clinical Oral Investigations 26 (2022): 4549–4558, 10.1007/s00784-022-04421-0. 35322316

[15] K. F. Schulz , D. G. Altman , D. Moher , and Group C , “CONSORT 2010 Statement: Updated Guidelines for Reporting Parallel Group Randomised Trials,” International Journal of Surgery 9, no. 8 (2011): 672–677, 10.1016/j.ijsu.2011.09.004. 22019563

[16] T. Berglundh , G. Armitage , M. G. Araujo , et al., “Peri‐Implant Diseases and Conditions: Consensus Report of Workgroup 4 of the 2017 World Workshop on the Classification of Periodontal and Peri‐Implant Diseases and Conditions,” Journal of Periodontology 89, no. S1 (2018): S313–S318, 10.1002/JPER.17-0739. 29926955

[17] A. Monje , R. Pons , and P. Pena , “Electrolytic Surface Decontamination in the Reconstructive Therapy of Peri‐Implantitis: Single‐Center Outcomes,” International Journal of Periodontics & Restorative Dentistry 45, no. 2 (2025): 185–198, 10.11607/prd.7151. 38820275

[18] A. Monje , R. Pons , A. Insua , J. Nart , H. L. Wang , and F. Schwarz , “Morphology and Severity of Peri‐Implantitis Bone Defects,” Clinical Implant Dentistry and Related Research 21, no. 4 (2019): 635–643, 10.1111/cid.12791. 31087457

[19] A. Ramanauskaite , F. Schwarz , E. A. Cafferata , and P. Sahrmann , “Photo/Mechanical and Physical Implant Surface Decontamination Approaches in Conjunction With Surgical Peri‐Implantitis Treatment: A Systematic Review,” Journal of Clinical Periodontology 50, no. S26 (2023): 317–335, 10.1111/jcpe.13783. 36709953

[20] V. T. Stuani , D. M. Kim , M. Nagai , C. Y. Chen , and A. C. P. Sant'Ana , “Effectiveness and Surface Changes of Different Decontamination Protocols at Smooth and Minimally Rough Titanium Surfaces,” Journal of Periodontology 92, no. 5 (2021): 704–715, 10.1002/JPER.20-0324. 32946119

[21] A. Monje , R. Pons , J. Vilarrasa , J. Nart , and H. L. Wang , “Significance of Barrier Membrane on the Reconstructive Therapy of Peri‐Implantitis: A Randomized Controlled Trial,” Journal of Periodontology 94, no. 3 (2023): 323–335, 10.1002/JPER.22-0511. 36399349

[22] A. Monje and J. Nart , “Disease Recurrence During Supportive Therapy Following Peri‐Implantitis Treatment: A Retrospective Study,” Journal of Periodontal Research 59, no. 5 (2024): 918–928, 10.1111/jre.13281. 38693780

[23] A. M. Roos‐Jansaker , H. Renvert , C. Lindahl , and S. Renvert , “Submerged Healing Following Surgical Treatment of Peri‐Implantitis: A Case Series,” Journal of Clinical Periodontology 34, no. 8 (2007): 723–727, 10.1111/j.1600-051X.2007.01098.x. 17535286

[24] J. T. Lee , I. S. Jang , J. H. Moon , and S. M. Yang , “In Vitro Evaluation of Implant Surface Decontamination Methods Based on Removal and Regrowth of Microorganisms,” International Journal of Oral & Maxillofacial Implants 36, no. 6 (2021): 1088–1094, 10.11607/jomi.8878. 34919605

[25] S. Schneider , M. Rudolph , V. Bause , and A. Terfort , “Electrochemical Removal of Biofilms From Titanium Dental Implant Surfaces,” Bioelectrochemistry 121 (2018): 84–94, 10.1016/j.bioelechem.2018.01.008. 29413867

[26] M. Schlee , H. L. Wang , T. Stumpf , U. Brodbeck , D. Bosshardt , and F. Rathe , “Treatment of Periimplantitis With Electrolytic Cleaning Versus Mechanical and Electrolytic Cleaning: 18‐Month Results From a Randomized Controlled Clinical Trial,” Journal of Clinical Medicine 10, no. 16 (2021): 3475, 10.3390/jcm10163475. 34441770 PMC8397046

[27] F. Gianfreda , A. Punzo , V. Pistilli , et al., “Electrolytic Cleaning and Regenerative Therapy of Peri‐Implantitis in the Esthetic Area: A Case Report,” European Journal of Dentistry 16, no. 4 (2022): 950–956, 10.1055/s-0042-1750773. 35785819 PMC9683897

[28] S. Bernardi , E. Qorri , G. Botticelli , et al., “Use of Electrical Field for Biofilm Implant Removal,” European Review for Medical and Pharmacological Sciences 27, no. 3S (2023): 114–121, 10.26355/eurrev_202304_31328. 37129321

[29] F. J. González , E. Requena , L. Miralles , et al., “Adjuvant Effect of Titanium Brushes in Peri‐Implant Surgical Treatment: A Systematic Review,” Dentistry Journal 9, no. 8 (2021): 84, 10.3390/dj9080084. 34435996 PMC8393649

[30] I. Sanz‐Martín , K. Paeng , H. Park , J. K. Cha , U. W. Jung , and M. Sanz , “Significance of Implant Design on the Efficacy of Different Peri‐Implantitis Decontamination Protocols,” Clinical Oral Investigations 25, no. 6 (2021): 3589–3597, 10.1007/s00784-020-03681-y. 33170374

[31] V. Steiger‐Ronay , A. Merlini , D. B. Wiedemeier , P. R. Schmidlin , T. Attin , and P. Sahrmann , “Location of Unaccessible Implant Surface Areas During Debridement in Simulated Peri‐Implantitis Therapy,” BMC Oral Health 17, no. 1 (2017): 137, 10.1186/s12903-017-0428-8. 29183313 PMC5706147

[32] G. A. Kotsakis and S. M. Ganesan , “Microbial Dysbiosis, Titanium Release, and Peri‐Implantitis,” Journal of Dental Research 104, no. 5 (2025): 473–480, 10.1177/00220345241307939. 39953673 PMC12310141

[33] G. A. Kotsakis , R. Black , J. Kum , et al., “Effect of Implant Cleaning on Titanium Particle Dissolution and Cytocompatibility,” Journal of Periodontology 92, no. 4 (2021): 580–591, 10.1002/JPER.20-0186. 32846000

[34] A. Mombelli , A. Feloutzis , U. Bragger , and N. P. Lang , “Treatment of Peri‐Implantitis by Local Delivery of Tetracycline. Clinical, Microbiological and Radiological Results,” Clinical Oral Implants Research 12, no. 4 (2001): 287–294, 10.1034/j.1600-0501.2001.012004287.x. 11488856

[35] A. Alonso‐Espanol , E. Bravo , A. Carrillo de Albornoz , et al., “Antimicrobial Effect and Cytocompatibility After Using Different Decontamination Methods on Titanium Implant Surfaces: An In Vitro Study,” Clinical Oral Implants Research 36 (2025): 626–639, 10.1111/clr.14410. 39878350

[36] M. Gosau , S. Hahnel , F. Schwarz , T. Gerlach , T. E. Reichert , and R. Burgers , “Effect of Six Different Peri‐Implantitis Disinfection Methods on In Vivo Human Oral Biofilm,” Clinical Oral Implants Research 21, no. 8 (2010): 866–872, 10.1111/j.1600-0501.2009.01908.x. 20666798

[37] K. Nakamura , M. Shirato , T. Tenkumo , et al., “Hydroxyl Radicals Generated by Hydrogen Peroxide Photolysis Recondition Biofilm‐Contaminated Titanium Surfaces for Subsequent Osteoblastic Cell Proliferation,” Scientific Reports 9, no. 1 (2019): 4688, 10.1038/s41598-019-41126-z. 30886168 PMC6423011

[38] M. Alhag , S. Renvert , I. Polyzois , and N. Claffey , “Re‐Osseointegration on Rough Implant Surfaces Previously Coated With Bacterial Biofilm: An Experimental Study in the Dog,” Clinical Oral Implants Research 19, no. 2 (2008): 182–187, 10.1111/j.1600-0501.2007.01429.x. 18039336

[39] S. G. Kolonidis , S. Renvert , C. H. Hämmerle , N. P. Lang , D. Harris , and N. Claffey , “Osseointegration on Implant Surfaces Previously Contaminated With Plaque. An Experimental Study in the Dog,” Clinical Oral Implants Research 14, no. 4 (2003): 373–380, 10.1034/j.1600-0501.2003.01871.x. 12868999

[40] M. Bassetti , D. Schar , B. Wicki , et al., “Anti‐Infective Therapy of Peri‐Implantitis With Adjunctive Local Drug Delivery or Photodynamic Therapy: 12‐Month Outcomes of a Randomized Controlled Clinical Trial,” Clinical Oral Implants Research 25, no. 3 (2014): 279–287, 10.1111/clr.12155. 23560645

[41] A. Monje , R. Pons , E. Amerio , H. L. Wang , and J. Nart , “Resolution of Peri‐Implantitis by Means of Implantoplasty as Adjunct to Surgical Therapy: A Retrospective Study,” Journal of Periodontology 93, no. 1 (2022): 110–122, 10.1002/JPER.21-0103. 33904175

[42] K. Jepsen , S. Jepsen , M. L. Laine , et al., “Reconstruction of Peri‐Implant Osseous Defects: A Multicenter Randomized Trial,” Journal of Dental Research 95, no. 1 (2016): 58–66, 10.1177/0022034515610056. 26450511

[43] N. Pranno , M. P. Cristalli , F. Mengoni , et al., “Comparison of the Effects of Air‐Powder Abrasion, Chemical Decontamination, or Their Combination in Open‐Flap Surface Decontamination of Implants Failed for Peri‐Implantitis: An Ex Vivo Study,” Clinical Oral Investigations 25, no. 5 (2021): 2667–2676, 10.1007/s00784-020-03578-w. 32975703 PMC8060238

[44] A. Leonhardt , G. Dahlen , and S. Renvert , “Five‐Year Clinical, Microbiological, and Radiological Outcome Following Treatment of Peri‐Implantitis in Man,” Journal of Periodontology 74, no. 10 (2003): 1415–1422, 10.1902/jop.2003.74.10.1415. 14653386

[45] A. Monje , R. Pons , A. Roccuzzo , G. E. Salvi , and J. Nart , “Reconstructive Therapy for the Management of Peri‐Implantitis via Submerged Guided Bone Regeneration: A Prospective Case Series,” Clinical Implant Dentistry and Related Research 22, no. 3 (2020): 342–350, 10.1111/cid.12913. 32410379

[46] Y. Zhu , Y. Xu , Z. Ling , C. Zhao , A. Xu , and F. He , “The Biofilm Removal Effect and Osteogenic Potential on the Titanium Surface by Electrolytic Cleaning: An In Vitro Comparison of Electrolytic Parameters and Five Techniques,” Clinical Oral Implants Research 35, no. 4 (2024): 454–466, 10.1111/clr.14245. 38345170

[47] M. A. Assuncao , J. Botelho , V. Machado , et al., “Dental Implant Surface Decontamination and Surface Change of an Electrolytic Method Versus Mechanical Approaches: A Pilot In Vitro Study,” Journal of Clinical Medicine 12, no. 4 (2023): 1703, 10.3390/jcm12041703. 36836238 PMC9967341

[48] A. Monje , R. Pons , J. Nart , R. J. Miron , F. Schwarz , and A. Sculean , “Selecting Biomaterials in the Reconstructive Therapy of Peri‐Implantitis,” Periodontology 2000 94, no. 1 (2024): 192–212, 10.1111/prd.12523. 37728141

[49] A. Aghazadeh , G. Rutger Persson , and S. Renvert , “A Single‐Centre Randomized Controlled Clinical Trial on the Adjunct Treatment of Intra‐Bony Defects With Autogenous Bone or a Xenograft: Results After 12 Months,” Journal of Clinical Periodontology 39, no. 7 (2012): 666–673, 10.1111/j.1600-051X.2012.01880.x. 22548359

[50] D. Buser , I. Urban , A. Monje , M. F. Kunrath , and C. Dahlin , “Guided Bone Regeneration in Implant Dentistry: Basic Principle, Progress Over 35 Years, and Recent Research Activities,” Periodontology 2000 93, no. 1 (2023): 9–25, 10.1111/prd.12539. 38194351

[51] I. A. Urban and A. Monje , “Guided Bone Regeneration in Alveolar Bone Reconstruction,” Oral and Maxillofacial Surgery Clinics of North America 31, no. 2 (2019): 331–338, 10.1016/j.coms.2019.01.003. 30947850

[52] E. Regidor , A. Ortiz‐Vigon , M. Romandini , C. Dionigi , J. Derks , and M. Sanz , “The Adjunctive Effect of a Resorbable Membrane to a Xenogeneic Bone Replacement Graft in the Reconstructive Surgical Therapy of Peri‐Implantitis: A Randomized Clinical Trial,” Journal of Clinical Periodontology 50, no. 6 (2023): 765–783, 10.1111/jcpe.13796. 36802084

[53] A. Turri , I. Elgali , F. Vazirisani , et al., “Guided Bone Regeneration Is Promoted by the Molecular Events in the Membrane Compartment,” Biomaterials 84 (2016): 167–183, 10.1016/j.biomaterials.2016.01.034. 26828682

[54] A. Monje and F. Schwarz , “Principles of Combined Surgical Therapy for the Management of Peri‐Implantitis,” Clinical Advances in Periodontics 12, no. 1 (2022): 57–63, 10.1002/cap.10186. 34569711

