# Supplementary material for: Surface Decontamination on the Reconstructive Therapy of Peri‐Implantitis: A Multicenter Randomized Clinical Trial
Source: Clin Implant Dent Relat Res. 2025 Jul 22;27(4):e70075. doi: 10.1111/cid.70075 (PMC12281608; doi:10.1111/cid.70075)
Supplement: Supplementary file 1 — Data S1. [file CID-27-0-s002.pdf]

APROVACIÓ ESTUDI PEL CEIC / APROBACIÓN ESTUDIO POR EL CEIC / RESEARCH ETHICAL COMMITTEE APPROVAL STUDY

Codi de l'estudi/Código del estudio/Study Code: PER-ECL-2022-08
Títol/Título/Title: Influence of Surface Detoxification Strategy on The Surgical Reconstructive Therapy of Peri-implantitis: Multi-center Randomized Controlled Trial
Protocol/o: Versió/n 3.0 *Data/Fecha/Version date:* 22/02/23
Investigador/a Secundari/a/ Researchers: Dr. Joan Pi, Ramón Pons
Tutor/Monitor: Dr. Alberto Monje
Investigadors/es Principals/es/Main researchers: Dr. José Nart

Sant Cugat del Vallès, 15 de març de 2023

Benvolgut Investigador/a,

Els membres del CEIm de la Clínica Universitària d'Odontologia de la UIC, els hi agraeixen l'aportació científica en el camp de la investigació i la presentació del Protocol en aquest Comitè per a la seva avaluació.

Valorades les noves aportacions realitzades a l'estudi, sol·licitades pel nostre CEIm, el dia 22 de febrer de 2023, li comuniquem que el dictamen final ha sigut FAVORABLE en la reunió del dia 08/03/23, acta 8AG 08/03/23

Li informem que s'haurà de presentar al Comitè d'Ètica d'investigacions amb medicaments, i a través de la Comissió Científica, un informe preliminar anual del seguiment de l'estudi i un informe final un cop finalitzat aquest.

El Comitè, tant en la seva composició, com en els PNT, compleix amb les normes de BPC (CPMP/ICH/135/95) i amb el Real Decret 1090/2015, i la seva composició actual és la següent:

- Dr. J.Manuel Ribera Uribe (Presidente, Medico-estomatólogo)
- Dr. Pau Ferrer Salvans (Vicepresidente, Farmacólogo clínico)
- Sra. Noelia Nogales (Secretaria técnica, Bióloga)
- Dr. Joan Janáriz Roldán (Miembro, Médico especialista en medicina interna i oncología)
- Dr. Andreu Hernando Chaure (Miembro, Jurista)
- Sra. Cristina Roure Nuez (Miembro, Farmacéutica Hospitalaria)
- Sra. Klaudia Oboloñczyk (Miembro, Farmacéutica de Atención Primaria)
- Dr. Dr. Josep Argemí Renom (Miembro, Médico pediatra)
- Sra. Laia Wennberg Capellades (Miembro, Enfermera)
- Sr. Antonio Alcáraz Gibert (Miembro lego, Persona ajena a la profesión sanitaria)
- M. Carmen García Monge (Miembro, Unidad de atención al usuario)
- David Gómez Zaragoza (Miembro, Delegado de protección de datos)
- Dra. Rosa María Cabanas (Miembro, Fisioterapeuta)
- Dr. Jose Angel Delgado Garcia-Menocal (Miembro, Químico)

Que en aquesta reunió del Comitè Ètic d'Investigació Clínica amb medicaments es va cumplir amb el quorum preceptiu legalment.

Atentament,

Apreciados Doctores,

Los miembros del CEIm de la Clínica Universitària d'Odontologia de la UIC, les agradecen su aportación científica en el campo de la investigación y la presentación del Protocolo a este Comité para su evaluación.

Valoradas las nuevas aportaciones realizadas al estudio, solicitadas por nuestro CEIm, el 22 de febrero de 2023, le comunicamos que el dictamen final ha sido FAVORABLE, en la reunión del día 08/03/23, acta 8AG 08/03/23.

Le recordamos que deberá presentar al Comitè d'Ètica d'Investigacions amb medicaments de la CUO, y a través de la Comisión Científica, un informe preliminar mensual del seguimiento del estudio y un informe final una vez finalizado el mismo.

Atentamente,

Dear Doctors,

The members of the CEIm of the Clínica Universitària d'Odontologia appreciate your contribution in the field of research and the presentation to this Committee of the referred study for its evaluation.

After having rated the new contributions to the study, requested by our Ethic Committee, on 22/02/23, the decision was to APPROVE it at the meeting of acta 8AG 08/03/23.

We remind that you should present a monthly preliminary report during the study and a final report when the study finishes, through the Academic Commission, to the Drug Research Ethics Committee.

Best regards,

RIBERA URIBE JOSE MANUEL - 37314164E

Firmado digitalmente por RIBERA URIBE JOSE MANUEL - 37314164E
Fecha: 2023.03.16 08:40:30 +01'00'

Dr. J.Manuel Ribera
President CEIm
