# Supplementary material for: Surface Decontamination on the Reconstructive Therapy of Peri‐Implantitis: A Multicenter Randomized Clinical Trial
Source: Clin Implant Dent Relat Res. 2025 Jul 22;27(4):e70075. doi: 10.1111/cid.70075 (PMC12281608; doi:10.1111/cid.70075)
Supplement: Supplementary file 2 — Data S2. [file CID-27-0-s004.pdf]

Gerencia del
Area de Salud
de Badajoz
Comité de Ética de la Investigación

JUNTA DE EXTREMADURA
Consejería de Sanidad y Servicios Sociales

D. JULIO BENITEZ RODRIGUEZ, SECRETARIO DEL COMITÉ DE ÉTICA DE LA INVESTIGACIÓN DE BADAJOZ"

CERTIFICA:

Que este Comité ha analizado la solicitud de evaluación del proyecto de investigación: *"INFLUENCIA DE LA ESTRATEGIA DE DETOXIFICACIÓN DE LA SUPERFICIE PARA LA TERAPIA RECONSTRUCTIVA DE LA PERI-IMPLANTITIS: ESTUDIO MULTI-CÉNTRICO ALEATORIZADO"*, para su realización en el Centro de Implantología Cirugía Oral y Maxilofacial de Badajoz, siendo el Investigador Principal D. Alberto Monje Correa.

Y mediante este documento expresa que este proyecto se ajusta a las normas éticas esenciales utilizadas como referencia en este ámbito y ha decidido por unanimidad expresar la idoneidad científica del proyecto, así como su apoyo al mismo emitiendo DICTAMEN FAVORABLE.

Lo que Certifico para su conocimiento y efectos en Badajoz a 31 de agosto de 2022.

SERVICIO EXTREMEÑO DE SALUD
Comité Ético de la Investigación
CEI
BADAJOZ
