# Supplementary material for: Surface Decontamination on the Reconstructive Therapy of Peri‐Implantitis: A Multicenter Randomized Clinical Trial
Source: Clin Implant Dent Relat Res. 2025 Jul 22;27(4):e70075. doi: 10.1111/cid.70075 (PMC12281608; doi:10.1111/cid.70075)
Supplement: Supplementary file 3 — Data S3. [file CID-27-0-s001.pdf]

04/2021

Dr. Dionisio Lorenzo Lorenzo Villegas, presidente del Comité de Bioética de la Universidad Fernando Pessoa Canarias,

C E R T I F I C A:

Que, con respecto a la propuesta de estudio de Alberto Monje para la realización del estudio de Investigación abajo señalado y aprobado «con informe favorable condicionado a la subsanación de defectos formales o a la aportación de la documentación adicional expresamente solicitada», con fecha 19 de noviembre, los documentos de subsanación de defectos formales y la documentación adicional expresamente solicitada aportados por el investigador que hace la propuesta de estudio resultan pertinentes, adecuados y suficientes para la obtención de aprobado por este comité.

"Título: Influence of Surface Detoxification Strategy on The Surgical Reconstructive Therapy of Peri-implantitis: Multi-center Randomized Controlled Trial."

Lo que le traslado a los efectos oportunos.

Santa María de Guía, a 16 de diciembre de 2021

Dionisio L. Lorenzo Villegas

Firmado digitalmente por Dionisio L. Lorenzo Villegas
Fecha: 2021.12.16 19:33:49 Z

Presidente de Comité de Bioética de la UFPC

DIRECCIÓN EEPI

ACREDITACIÓN Y CALIDAD DE TÍTULO
