# Supplementary material for: Surface Decontamination on the Reconstructive Therapy of Peri‐Implantitis: A Multicenter Randomized Clinical Trial
Source: Clin Implant Dent Relat Res. 2025 Jul 22;27(4):e70075. doi: 10.1111/cid.70075 (PMC12281608; doi:10.1111/cid.70075)

**Supplementary figure 1.** Diagnostic accuracy of the equation formulated entailing marginal bone loss, surface decontamination modality and width of keratinized mucosa.


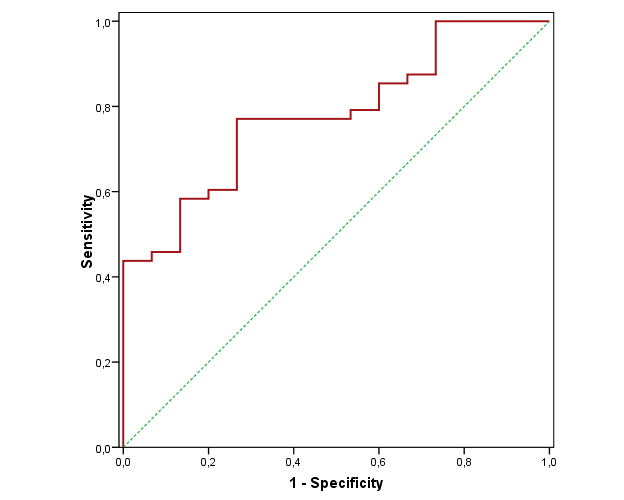

Supplement: Supplementary file 4 — Data S4. [file CID-27-0-s003.docx]
